# A lightweight network for phone surface defect detection with industrial deployment on RK3568 edge devices

**DOI:** 10.1371/journal.pone.0336971

**Published:** 2025-12-19

**Authors:** Shulong Zhuo, Xiaojian Zhou, Jiajing Cai, Hao Bai, Xu Duan, YiQun Ma

**Affiliations:** 1 School of Information Engineering, Hainan Vocational University of Science and Technology, Haikou, Hainan, China; 2 School of Information Science and Technology, HaiNan Normal University, Haikou, Hainan, China; The University of British Columbia, AUSTRALIA

## Abstract

In smartphone production, detecting small defects on screens remains challenging due to low detection accuracy, high missed detection rates, and slow processing speeds. To address these issues, this paper presents a Lightweight Network Based on YOLOv8 (LNB-YOLO) for defect detection, with several key enhancements. First, a Feature Pyramid Network based on Context-Guided Spatial Feature Reconstruction (CGRFPN) is integrated to improve the perception of multi-level features and enhance small target recognition in complex backgrounds. Second, the Efficient Local Attention (ELA) module is incorporated into the Backbone’s C2F module to improve localization precision, while the Minimum Point Distance based IoU (MPDIoU) loss function is employed to prevent gradient explosion. Third, a lightweight Detail-Enhanced Convolution and Shared Convolutional Detection Head (LSDECD) is designed to capture fine details while reducing parameters and computational complexity. Finally, model pruning and knowledge distillation techniques are applied to further optimize efficiency. Experimental results on the PKU-Market-Phone dataset show that LNB-YOLO achieves a mAP@0.5 of 97.5% and a mAP@.5:.95 of 68.8%, surpassing the original YOLOv8 by 6.1% and 9.3%, respectively. The model also reduces parameters by 80% and computational cost by 63%, effectively meeting precision requirements for smartphone production lines.

## Introduction

Mobile phone screens are susceptible to physical damage during production, resulting in defects such as oil stains, scratches, and discoloration, which lower production yields. As a result, defect detection has become a critical step in quality control. While traditional manual visual inspection can deliver accurate results, it is constrained by high labor costs, low efficiency, and subjective bias [1]. Automated detection methods driven by computer vision and deep learning technologies offer faster and more precise defect identification, significantly enhancing production efficiency and also leading to the development of numerous advanced algorithms and models [2].

In large-target defect detection, Peng et al. [3]. proposed a method for detecting surface defects on metal cabinets by segmenting boundary regions into equal-sized patches and establishing a Gaussian distribution model to identify anomalies, achieving superior detection performance. Similarly, Shang et al. [4]. introduced a Transformer-based defect-aware network where convolutional layers were replaced by a Transformer encoder, and a defect-aware module captured irregularly shaped features, demonstrating robust detection on blade and tool wear datasets. Recent research has increasingly focused on small-target defect detection, a challenging but essential task. Yang et al. [5]. proposed a deep learning algorithm based on a single-shot detector network to detect small defects in industrial components, effectively mitigating interference from conveyor speed and camera parameters. Although accurate, the method suffered from long inference times. Cao et al. [6]. presented the FusionScratchNet (FS-Net) defect detection model for cell phone screen scratches, FS-Net effectively captures both global and local features, thereby enhancing defect feature representation. Despite promising detection results, the model faced challenges related to inference latency and deployment at the edge.

Given the growing demand for edge-deployable models, YOLO-based object detection algorithms have gained attention. For example Jing et al. [7]. improved YOLOv3 for fabric defect detection by incorporating k-means clustering to better detect defects in gray and mesh fabrics. However, the model exhibited poor generalization and high computational complexity. Le et al. [8]. introduced an enhanced YOLOv5 algorithm for industrial surface defects, utilizing a BiFPN to reduce missed and false detections but at the expense of increased detection complexity and slower inference speeds. Wang et al. [9]. optimized the YOLOv8 spatial pyramid pooling layer using a SimSPPF module and integrated BiFPN with the LSK dynamic large-kernel attention mechanism, achieving improved detection accuracy for small targets. However, the model neglected considerations for edge deployment.

To address challenges in smartphone surface defect detection, this paper proposes the LNB-YOLO model, built on the YOLOv8 framework with several key optimizations to improve detection performance:

**Enhanced feature extraction:**

1) The Context-Guided Reconstruction Feature Pyramid Network (CGRFPN) is introduced in the head, incorporating the Rectangular Self-Calibration Module (RCM), which integrates Rectangular Self-Calibration Attention (RCA) and a lightweight multilayer perceptron (MLP) decoder, improving focus on shallow features and accelerating recognition [10].

2) The Pyramid Context Extraction (PCE) module enhances contextual awareness by consolidating features at different levels.

3) Multi-scale features are fused using the Multi-Feature Fusion Module (MFF), Dynamic Interpolation Fusion Module (DIF), and Get Index Output (GIO), boosting multi-scale feature representation and recognition.

**Backbone optimization:** The Efficient Local Attention (ELA) module is integrated into the C2F module, employing average pooling to extract horizontal and vertical feature vectors, thereby enhancing the detection precision for small defects [11].

**Enhanced loss function:** The adoption of the MPDIoU loss function prevents gradient explosion and ensures stable training with improved accuracy [12].

**Lightweight detection layer:** A custom-designed LSDECD lightweight detection head captures fine details while reducing parameter size and computational complexity [13].

**Pruning and knowledge distillation:** Pruning and knowledge distillation are applied to further reduce model complexity and computational load, making it suitable for deployment on edge devices [14].

These comprehensive optimizations enable the INB-YOLO model to achieve superior detection accuracy and computational efficiency compared to existing YOLO-based methods, making it well-suited for real-world smartphone production scenarios and edge computing deployments.

The structure of the paper is structured as follows:

**Introduction:** Provides an overview of advancements and limitations in defect detection and introduces the proposed LNB-YOLO algorithm.

**Related work:** Introduces the current state and challenges of object detection methods, and discusses the trend of lightweight design and edge deployment as the future direction of development in the field of object detection.

**Proposed methodology:** Describes the architecture, optimization strategies, and implementation of the LNB-YOLO model.

**Experimental setup and performance analysis:** Covers experimental conditions, datasets, and evaluation metrics. It also discusses the performance improvements of the LNB-YOLO model through ablation studies, lightweight design, pruning, and knowledge distillation, with comparisons to other models.

**Deployment and generalization:** Demonstrates the deployment process and evaluates the generalization capability of the LNB-YOLO model.

**Conclusion and future work:** Summarizes the paper’s contributions and suggests directions for future research.

## Relate work

### Object detection methods

Object detection can be classified into two main categories: traditional methods and deep learning methods [15]. Traditional object detection methods primarily rely on manually designed feature extraction techniques, such as Histogram of Oriented Gradients(HOG) [16], Deformable Part Models(DPM) [17], Local Binary Patterns(LBP) [18], ColorNames, and Scale-Invariant Feature Transform(SIFT) [19]. These methods perform well in certain specific scenarios but are limited in performance on large-scale datasets due to the manually designed feature extractors and classifiers. To overcome the limitations of traditional methods, deep learning approaches were introduced into the field of object detection. Deep learning-based object detection methods are generally divided into two categories: two-stage and one-stage object detection methods. Among them, R-CNN [20] and its improved versions, Fast R-CNN and Faster R-CNN, are representative models of two-stage object detection. R-CNN first generates candidate regions using selective search and then extracts features from these regions using a Convolutional Neural Network(CNN), followed by classification using a Support Vector Machine(SVM). Fast R-CNN [21] and Faster R-CNN [22] are further optimizations of R-CNN, improving detection speed and accuracy by sharing convolutional features and introducing Region Proposal Networks(RPN). However, two-stage models experience a significant drop in detection performance when faced with complex image backgrounds and small targets [23]. Additionally, two-stage detection algorithms are complex, have a large number of parameters, and are slow in inference. Therefore, one-stage detection algorithms are generally preferred in industrial applications, with YOLO being one of the representative models.

You Only Look Once (YOLO) [24] is a real-time object detection algorithm that predicts object categories and bounding boxes in a single forward pass, achieving end-to-end detection. Its strengths are high speed and simple structure. However, YOLO struggles with small objects, densely arranged targets, and demands high computational resources, limiting edge deployment. Thus, in industrial applications, optimization focuses on enhancing small-object detection accuracy and lightweighting the model for efficient deployment on edge devices [25].

### Lightweight design and edge deployment of object detection algorithms

As the application of object detection in industrial scenarios continues to expand, the demand for deploying lightweight networks is increasing. Achieving model lightweighting while maintaining detection accuracy is crucial to meet the required standards [26]. Researchers are currently focused on designing CNN architectures with fewer parameters. For example, the SqueezeNet model uses the Fire module to reduce the number of parameters, achieving model compression. Later, models like MobileNet [27], ShuffleNet [28], and MicroNet [29] emerged, which perform well in terms of detection speed and accuracy in practical applications. Researchers also found that introducing lightweight architectures into object detection models to form hybrid models is an effective approach to achieving lightweighting. A typical case is the CNN–Vision Transformer (ViT) hybrid model, which is commonly used. ViTs excel at generating feature maps with global information, while lightweight CNNs are better at generating feature maps with local information. By introducing convolution layers into the ViTs architecture, hybrid networks are formed, achieving an excellent balance in terms of parameter size and FLOPs, as seen in models like MobileViT [30] and RT-DETR [31].

Lightweight structures can reduce the computational cost of the model but may increase memory access demands and consume significant resources, as seen with Depthwise Separable Convolutions(DWConv). DWConv performs convolution on each channel separately, achieving spatial filtering, and uses 1x1 pointwise convolutions to exchange information between channels and generate feature maps. However, the 1x1 convolutions involve significant computational overhead and memory resource consumption. Therefore, simply relying on the introduction of lightweight structures cannot completely solve the problem of object detection model lightweighting. To address the high deployment cost of models, model compression techniques are proposed. Key methods for model compression include pruning [32]. Model pruning reduces the model’s resource space usage by removing unimportant parts of the network, thereby improving inference efficiency. The approach is to identify a subset of weights such that the pruned model’s performance on the validation set is minimally affected. Thus, pruning is focused on identifying which parts of a pre-trained network can be pruned and ensuring that the model’s detection accuracy does not experience a drastic decline after pruning. The most effective method to avoid detection accuracy degradation is knowledge distillation [33]. The main process of knowledge distillation involves generating soft labels through a trained teacher model and using a loss function (such as Kullback-Leibler Divergence) to guide the training of the student model. This ensures that the student model’s output not only fits the real labels but also matches the soft labels generated by the teacher model. This results in a model that performs well in detection both before and after pruning and is easy to deploy on edge computing devices.

For example, Huang et al. [32]. proposed a lightweight network, YOLO-ULNet, and deployed it on embedded sensing devices to detect smoke and flames before forest fires occur. They used channel pruning and feature distillation methods to deploy the model on a Raspberry Pi(RPi) 4B, and the testing results met the real-time detection requirements for forest fire prevention. Chen et al. [34]. proposed the SF-Yolov8n model based on YOLOv8n, adding the P2 layer and introducing an improved lightweight C2f module in the YOLOv8 model, as well as optimizing the loss function. They also applied pruning to reduce the model size and improve inference speed. Experimental results showed that the SF-Yolov8n model is easily deployable on resource-constrained devices and outperforms other mainstream detection models in detecting surface defects on dental nails in the medical industry. Idama et al. [35]. also proposed the QATFP-YOLO object detection model, which enables the deployment of object detection algorithms on low-power end devices. To enhance the inference speed of the QATFP-YOLO model, the authors applied two optimized training strategies: model pruning to reduce model size while maintaining accuracy, and filter pruning to remove redundant parameters, further reducing memory usage and inference time. Experimental results showed that the QATFP-YOLO model, after model pruning, achieved excellent inference speed and met detection performance requirements on non-GPU devices.

## Proposed methodology

### CGRFPN structure design

**Fig 1:**
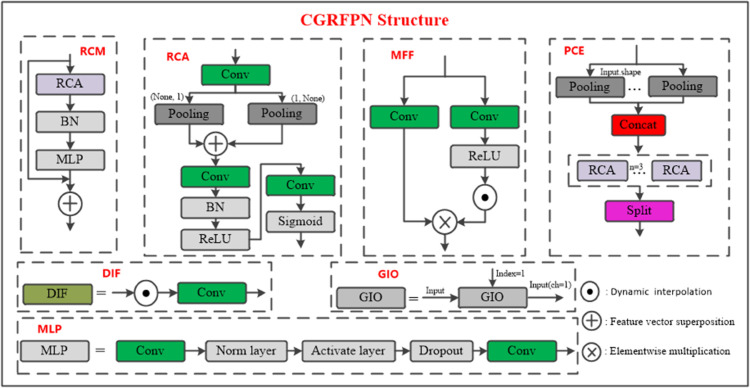
CGRFPN module structure. The image describes five key modules (PCE, MFF, RCM, DIF, GIO) to capture multi-scale features. It effectively fuses deep and shallow semantics through spatial reconstruction and dynamic interpolation, minimizing information loss and significantly improving object recognition accuracy.

The original YOLOv8 network’s FPN structure performs upsampling and downsampling on input feature maps, leading to the loss of shallow features. To address this, the LNB-YOLO network introduces the CGRFPN structure to achieve multi-scale feature extraction and effective feature output [10]. As illustrated in Fig 1.

**Fig 2:**
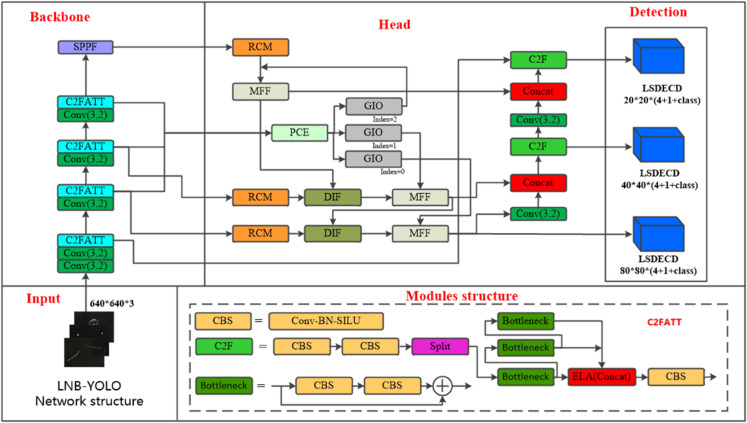
LNB-YOLO network structure. The image depicts the feature processing pipeline: the Backbone extracts initial features, which the RCM module processes via RCA for deep feature extraction. Subsequent DIF and MFF modules refine these into multi-scale representations. PCE and GIO then reconstruct features using dynamic interpolation. Finally, MFF further mines and fuses the reorganized features with other layers before passing them to the detection head.

The feature processing flow is shown in Fig 2. First, the Backbone extracts feature maps from the image, which are processed by the RCM module. Within RCM, the RCA component efficiently extracts deep features. These features are further refined through DIF and MFF to capture multi-scale information. The processed features are then passed to the PCE and GIO modules, where dynamic interpolation reconstructs deep features. Finally, the reorganized features undergo further mining by the MFF module and are fused with features from other layers before being fed into the detection head.

The CGRFPN structure effectively captures both deep and shallow features through spatial feature reconstruction and feature pyramids. The RCM and PCE modules focus on mining and fusing multi-scale features to prevent information loss. Meanwhile, the MFF, DIF and GIO modules leverage dynamic interpolation to enhance the model’s representation of multi-scale features, thereby improving target recognition capability.

### Add ELA attention mechanism

As illustrated in Fig 2, the Efficient Local Attention (ELA) mechanism is integrated into the C2FATT module within the Backbone of the LNB-YOLO model to enhance the detection of small defect targets on smartphone screens. This lightweight mechanism not only accurately captures regions of interest but also mitigates the problem of redundant information in feature channels.

**Fig 3:**
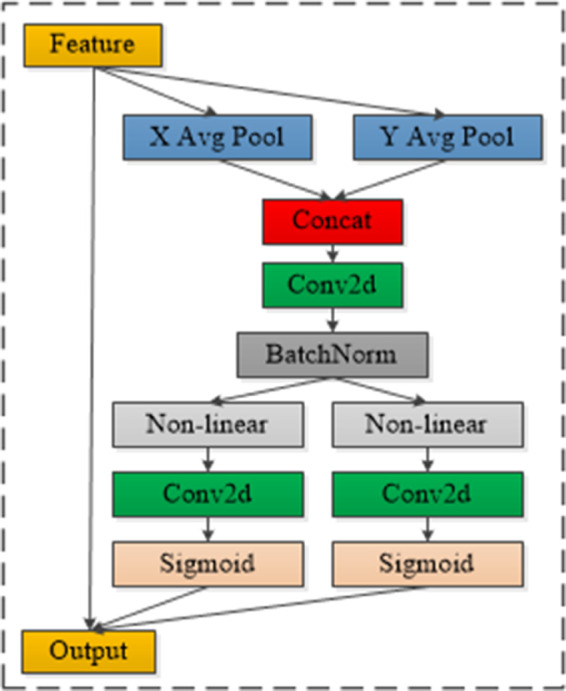
ELA module structure. The image illustrates the ELA mechanism. It employs average pooling along vertical and horizontal directions on image features to capture precise regions of interest while reducing channel redundancy, enhancing small defect detection in smartphone screens.

The structure of the ELA mechanism is depicted in Fig 3. Its operation involves performing average pooling on image features along the vertical and horizontal directions, with the pooling results mathematically defined by Eqs (1) and (2) [11].

$$z^{h}(h)=\frac{1}{W}\sum_{0\leq i\leq W}\mathrm{Avg}\left(x_{c}(h,i)\right)$$ (1)

$$z^{w}(w)=\frac{1}{H}\sum_{0\leq j\leq H}\mathrm{Avg}\left(x_{c}(j,w)\right)$$ (2)

In the formulas above, *z*^*h*^ represents the result of average pooling along the vertical direction, and *z*^*w*^ refers to the result of average pooling along the horizontal direction. The indices *h* and *w* denote the row and column positions of the feature matrix being pooled, while *H* and *W* represent the total number of rows and columns in the feature matrix. The variables i and j correspond to the current row and column values of the element being pooled, *x*_*c*_(*i*,*j*) indicates the original feature matrix, and Avg(·) refers to the average pooling function applied to the elements.

In the second step, the ELA mechanism performs feature fusion, convolution operations, and batch normalization on the averaged pooled features. This process is mathematically expressed in Eq (3), where BN represents the batch normalization operation, and ⊕ denotes the feature vector superposition operation.

$$f=\mathrm{BN}\left(z^{h}⊕z^{w}\right)$$ (3)

Subsequently, the ELA mechanism slices the batch-normalized features along the horizontal and vertical directions, transforming the feature matrix from dimensions C×H×W to C×H×1 and C×1×W, respectively. Non-linear activation and convolution operations are then applied. The processing workflow is mathematically expressed in Eq (4), where δ represents the non-linear activation function, and split(·) denotes the matrix slicing operation.

$$\left[f_{C\times H\times 1}^{h},f_{C\times 1\times W}^{w}\right]=\delta (\mathrm{split}(f))$$ (4)

Finally, the features $f_{C\times H\times 1}^{h}$ and $f_{C\times 1\times W}^{w}$ are individually activated using the sigmoid(·) function, followed by matrix multiplication. The resulting outputs are then element-wise multiplied with the original feature matrix *x*_*c*_(*i*,*j*) to produce the output of the ELA attention mechanism. The processing results are mathematically expressed in Eqs (5) and (6), where $g_{c}^{h}$ and $g_{c}^{w}$ are the results of the image features processed through the sigmoid(·) function. The ⊗ symbol represents the elementwise multiplication operation, and the symbol indicates the matrix multiplication operation.

$$g_{c}^{h}=\sigma \left(f_{C\times H\times 1}^{h}\right),\;g_{c}^{w}=\sigma \left(f_{C\times 1\times W}^{w}\right)$$ (5)

$$y_{c}(i,j)=x_{c}(i,j)⊗\left(g_{c}^{h}\cdot g_{c}^{w}\right)$$ (6)

**Fig 4:**
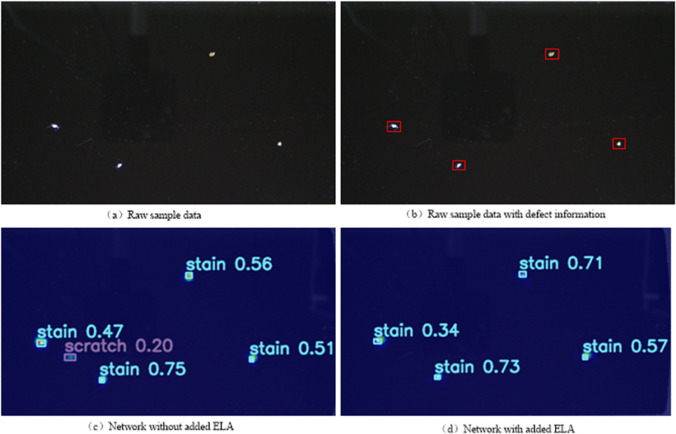
Heat map of ELA structure outputs. The image compares feature heatmaps with and without the ELA mechanism. The results demonstrate that ELA integration eliminates false positives for scratches while accurately detecting all stain defects, confirming its effectiveness in noise suppression and robustness enhancement for small target detection.

Fig 4 compares feature heatmaps before and after incorporating the ELA mechanism, using an image sample containing four stain defects. In Fig 4(c), the YOLOv8 model successfully identifies all four stain targets but generates false positives for scratch defects, failing to meet industrial standards. In contrast, Fig 4(d) shows the heatmap after integrating the ELA mechanism, where the network accurately detects all stain targets without any false positives. These results demonstrate that the ELA mechanism effectively suppresses noise and enhances the model’s robustness, significantly improving detection accuracy for small targets.

### LSDECD lightweight detection head design

In the YOLOv8 model, the detection head utilizes Batch Normalization (BatchNorm) for data normalization, which accelerates convergence, reduces dependence on initial weights, and mitigates overfitting risks. However, BatchNorm is highly sensitive to batch size; if the batch size is too small, the calculated mean and variance may not adequately represent the entire data distribution, leading to a decline in model performance. To address this issue, this study proposes a lightweight detection head based on a Detail-Enhanced Convolution (DEConv) module combined with a weight-sharing mechanism. The DEConv module comprises five parallel convolution layers: standard convolution (VC), central difference convolution (CDC), angular difference convolution (ADC), horizontal difference convolution (HDC), and vertical difference convolution (VDC). By incorporating differential convolution operations, the model’s representation capability and ability to handle unseen data are significantly enhanced. The mathematical expression for the DEConv module’s feature output is provided in Eq (7). It can be briefly understood as performing the five types of convolution operations on the input features and then applying elementwise multiplication to the results [13].

$$F_{out}=\mathrm{DEConv}\left(F_{in}\right)=\sum_{i=1}^{5}F_{in}*K_{i}=F_{in}*K_{cut}$$ (7)

In Eq (7), DEConv(·) represents the DEConv operation. *F*_*in*_ is the input feature at the current stage, and *K*_*i* = 1:5_ denotes the convolution kernels for VC, CDC, ADC, HDC, and VDC. The * symbol indicates the convolution operation, while *K*_*cut*_ refers to the transformed convolution kernels.

**Fig 5:**
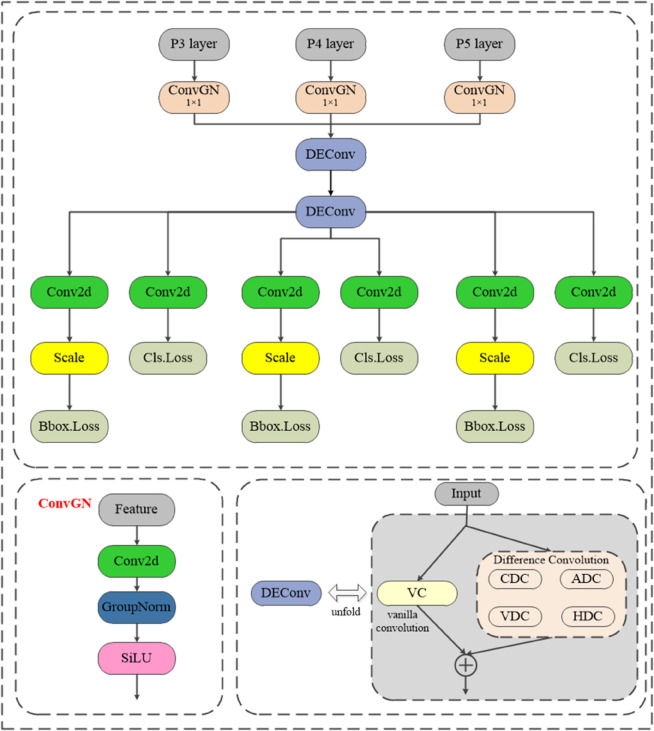
LSDECD structure diagram. The image shows the LSDECD module utilizes GroupNorm, parameter reduction, and DEConv to decrease batch dependency, improve efficiency, and enhance details.

Additionally, the weight-sharing mechanism reduces the model’s dependency on initial weights while also improving its inference speed [36]. As shown in Fig 5, the LSDECD structure encompasses the following three aspects:

**Group Normalization (GroupNorm):** Replacing Batch Normalization(BatchNorm) with GroupNorm to normalize each feature channel across training batches, thereby reducing the model’s dependency on batch size.

**Weight-Sharing Convolution Mechanism:** This mechanism reduces the number of parameters required for network training, improving computational efficiency and mitigating the risk of overfitting.

**Detail-Enhancement Convolution (DEConv):** Incorporating DEConv, which is equivalent to standard convolution in computational cost, while enhancing image detail information.

### Selection of loss function for LNB-YOLO network

In the original YOLOv8 network, the loss function employed is Complete Intersection Over Union(CIoU) [37], and its calculation is defined as follows:

$$IoU=\frac{B_{gt}\cap B}{B_{gt}\cup B}$$ (8)

$$L_{CIoU}=L_{IoU}+\frac{{\left(x-x_{gt}\right)}^{2}+{\left(y-y_{gt}\right)}^{2}}{W_{g}^{2}+H_{g}^{2}}+\alpha v$$ (9)

$$L_{IoU}=1-IoU=1-\frac{W_{i}H_{i}}{wh+w_{gt}h_{gt}-W_{i}H_{i}}$$ (10)

$$\alpha =\frac{v}{L_{IoU}+v}$$ (11)

$$v=\frac{4}{\pi^{2}}{\left(\arctan\frac{w}{h}-\arctan\frac{w_{gt}}{h_{gt}}\right)}^{2}$$ (12)

From the formulas mentioned above, it can be inferred that when the aspect ratios of the two bounding boxes are identical but their orientations differ, the aspect ratio correction term ν in CIOU becomes zero. This limitation causes the model to overlook directional discrepancies between bounding boxes.

To address this shortcoming, the MPDIoU loss function is proposed as an alternative. MPDIoU is a similarity measure for bounding boxes that calculates the minimum distance between the top-left and bottom-right corners of the predicted and ground truth boxes. The calculation formula is as follows [12]:

$$d_{1}^{2}={\left(x_{1}^{prd}-x_{1}^{gt}\right)}^{2}-{\left(y_{1}^{prd}-y_{1}^{gt}\right)}^{2}$$ (13)

$$d_{2}^{2}={\left(x_{2}{\;}^{prd}-x_{2}{\;}^{gt}\right)}^{2}-{\left(y_{2}{\;}^{prd}-y_{2}{\;}^{gt}\right)}^{2}$$ (14)

$$MPDIoU=IoU-\frac{d_{1}^{2}}{w^{2}+h^{2}}-\frac{d_{2}^{2}}{w^{2}+h^{2}}$$ (15)

In Eqs (13) and (14), $d_{1}^{2}$ and $d_{2}^{2}$ represent the squared Euclidean distances between the top-left and bottom right corners of the predicted and ground truth bounding boxes, respectively. These distances are used to quantify the positional offset between the boxes. Specifically, $x_{1}^{p}rd$, $y_{1}^{p}rd$ and $x_{2}^{p}rd$, $y_{2}^{p}rd$ denote the coordinates of the top-left and bottom-right corners of the predicted bounding box, while $x_{1}^{g}t$, $y_{1}^{g}t$ and $x_{2}^{g}t$, $y_{2}^{g}t$ correspond to the coordinates of the ground truth bounding box’s corners. In Eq (15), the IoU is computed as per Eq (8), where w and h represent the width and height of the input image.

These equations addresses the directional mismatch issue inherent in the CIOU loss function, thereby enhancing the model’s detection accuracy.

To evaluate the detection performance of the proposed network, four metrics were utilized: Positive Predictive Value (PPV), True Positive Rate (TPR), Average Precision (AP), and mean Average Precision (mAP). Specifically, mAP@0.5 denotes the mAP at an IoU threshold of 0.5, while mAP@.5:.95 is the mean mAP averaged over IoU thresholds of 0.5, 0.55, 0.6, 0.65, 0.7, 0.75, 0.8, 0.85, 0.9, and 0.95. The formulas for these metrics are as follows [38]:

$$PPV=\frac{TP}{TP+FP},\;TPR=\frac{TP}{TP+FN}$$ (16)

$$AP=\int_{0}^{1}p(r)dr,\;mAP=\frac{1}{n}\sum_{k=1}^{n}AP_{k}$$ (17)

$$FPR=\frac{FP}{TN+FP},\;FNR=\frac{FN}{TP+FN}$$ (18)

$$F1-\text{ score }=\frac{2\times PPV\times TPR}{PPV+TPR}$$ (19)

$$Macro-average=\frac{1}{K}\sum_{i=1}^{K}{\text{ index }}_{i}$$ (20)

- **TP (True Positive):** The number of positive samples correctly predicted as positive.
- **FP (False Positive):** The number of negative samples incorrectly predicted as positive.
- **FN (False Negative):** The number of positive samples incorrectly predicted as negative.
- **TN (True Negative):** The number of negative samples correctly predicted as negative.

### Pruning and knowledge distillation in the LNB-YOLO model

#### Unstructured model pruning algorithm.

The model pruning process consists of three main stages: model training, pruning, and fine-tuning. Model training involves pre-training a network to achieve satisfactory detection performance, although such models typically have a large number of parameters and high computational costs. Subsequently, a specific pruning method is applied to remove redundant information from the trained model, resulting in a more compact network with fewer parameters and faster inference speed [39]. However, this process may lead to a decline in detection accuracy. Finally, the pruned model undergoes fine-tuning and retraining to restore or even surpass the original model’s detection performance.

In the LNB-YOLO model, we employ the Layer-Adaptive Magnitude-based Pruning(LAMP) algorithm, a widely used pruning method in object detection. In neural networks, the weight of each connection determines the importance of input features in information transmission. Therefore, during pruning, the weight scoring mechanism determines whether a connection should be retained. In the Conv2D convolutional layers of the LSDECD detection head, the weight tensor is a four-dimensional matrix. To facilitate understanding, we flatten the four-dimensional weight tensor into a one-dimensional vector. Here, *u* and *v* represent weight indices, and *W*[*u*] and *W*[*v*] denote the weights at indices *u* and *v*, respectively. Assuming the flattened weights are sorted in ascending order, |W[v]|≥|W[u]| implies *v*>*u*. The score of a weight *W*[*u*] is defined as follows [40]:

$$\mathrm{score}(u;W)=\frac{(W[u]{)}^{2}}{\sum_{v\geq u}(W[v]{)}^{2}}$$ (21)

In this formula, the numerator represents the squared magnitude of the target weight, while the denominator is the sum of the squared magnitudes of all remaining weights in the same layer. This calculation measures the importance of a given weight relative to all other weights within the same layer. Higher-weight values correspond to higher scores, while lower-score weights are considered less critical and are pruned based on a predefined threshold.

**Fig 6:**
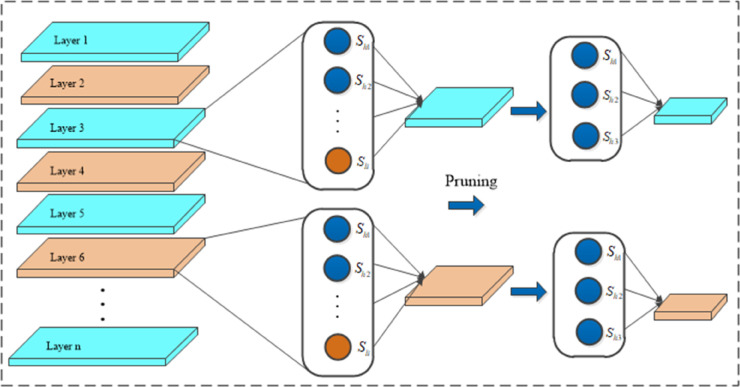
The schematic diagram of the LAMP algorithm. During pruning, to prevent complete layer removal and maintain network stability, each layer retains at least one connection with a score of 1, ensuring no layer loses all functionality.

During pruning, if all structural units within a layer are removed, the layer may lose functionality. To prevent this, each layer retains at least one connection with a score of 1, ensuring that no entire layer is pruned away, thereby maintaining the model’s stability and preventing network collapse, as shown in Fig 6.

#### Knowledge distillation algorithm.

Knowledge distillation is a model compression technique that involves training a high-performance yet computationally complex teacher model to guide a lightweight student model. The teacher model transfers knowledge to the student model, enabling it to learn richer feature representations, thereby maintaining model compactness while achieving detection performance comparable to or even exceeding that of the teacher model. Feature distillation, a specific form of knowledge distillation, optimizes the student model by transferring intermediate-layer features from the teacher model. By learning detailed information and contextual knowledge, the student model enhances its representation capability and generalization performance [41].

**Fig 7:**
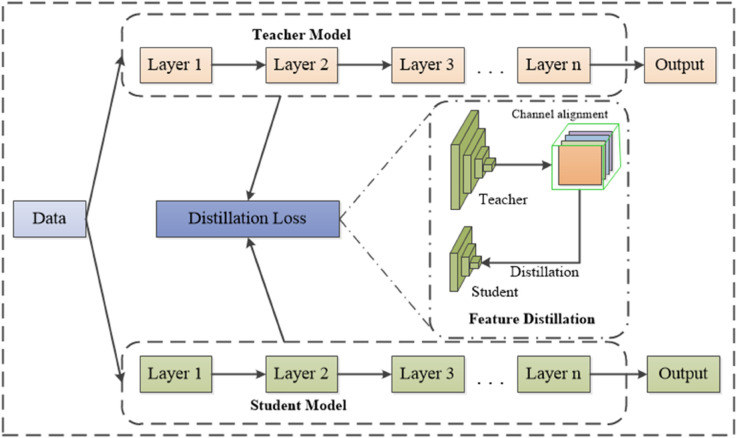
The feature distillation algorithm. The image illustrates the feature distillation algorithm in LNB-YOLO, which minimizes inter-network feature channel differences by reducing KL divergence to balance model compression and performance.

In the LNB-YOLO model, this method is employed to achieve an optimal balance between model compression and performance optimization. The feature distillation algorithm is illustrated in Fig 7, where the key objective is to minimize the differences between feature channels of the two networks as much as possible, such as by reducing the Kullback-Leibler (KL) Divergence value. To achieve this, the intermediate feature layers of both networks must first be transformed into probability distributions, as expressed in Eq (22) [42]:

$$\phi \left(y_{c}\right)=\frac{\exp\left(\frac{y_{c,i}}{\;T}\right)}{\sum_{i=1}^{W\times H}\exp\left(\frac{y_{c,i}}{\;T}\right)}$$ (22)

where *y*_*c*_ represents the feature values of the current network layer, *T* is the distillation temperature (a hyper-parameter), *W* and *H* denote the width and height of the feature map, respectively, and *i* is the index of the feature position in the feature space. ϕ(·) is the function that converts feature values into probability distributions.

The KL divergence between the two networks is computed using Eq (23) [42]:

$$\varphi \left(y^{Tea},y^{Stu}\right)=\frac{T^{2}}{C}\sum_{c=1}^{C}\sum_{i=1}^{W\times H}\phi \left(y_{c,i}^{Tea}\right)\cdot \log\left[\frac{\phi \left(y_{c,i}^{Tea}\right)}{\phi \left(y_{c,i}^{Stu}\right)}\right]$$ (23)

where $y_{c,i}^{Tea}$ and $y_{c,i}^{Stu}$ represent the feature values of the current layer in the teacher and student networks, respectively, and *c* is the channel index of the network, with c=1,2,…,C. The function $\varphi (y^{Tea},y^{Stu})$ evaluates the difference between the probability distributions from the teacher and student networks. A smaller KL divergence indicates that the student model effectively mimics the teacher model, ensuring a successful distillation process.

Finally, the total distillation loss for the student model is expressed in Eq (24):

$$L_{dl}=\sum_{i=1}^{n}(y^{Tea},y^{Stu})$$ (24)

where *n* represents the number of feature layers in the network, and $\varphi_{i}\left(y^{Tea},y^{Stu}\right)$ denotes the KL divergence of the corresponding feature layers in both models.

## Experimental setup and performance analysis

### Experimental setup and the dataset

#### Experimental setup.

The experiments were conducted on a server configured with 128GB of memory, an NVIDIA GeForce RTX 3090 GPU (24GB), and an AMD CPU. The deep learning framework used was PyTorch. The network was trained for 300 epochs, with a 3-epoch warm-up phase. The initial learning rate was set to 0.01, the weight decay coefficient to 0.0005, the batch size (Batch Size) to 16, and the optimizer momentum to 0.937. The input image resolution was fixed at 640×640.

#### The dataset of mobile surface defect.

The dataset used for training and testing was the PKU-Market-Phone dataset, publicly available from the Intelligent Robotics Open Lab at Peking University. This dataset consists of mobile phone screen surface defect images captured by industrial cameras in manufacturing environments. All images have a resolution of 1920×1080 pixels and cover three types of surface defects:

- **Oil Stains (Oil)**
- **Scratches (Scratch)**
- **Spots (Stain)**

**Fig 8:**
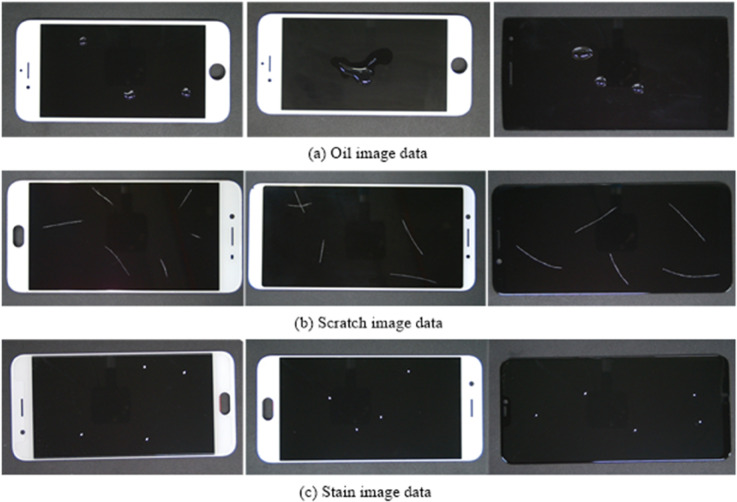
Display of defect samples in The PKU-Market-Phone dataset. The image shows representative samples from a 1,800-image dataset used for defect detection. The dataset, expanded via cropping, rotation, and scaling, is split into training, validation, and test sets in a 6:2:2 ratio.

To enhance the model’s adaptability to diverse defect scenarios, data augmentation techniques such as random cropping, image rotation, and scaling were employed to expand the dataset. The final dataset comprised **1,800 images**, each annotated with defect positions and categories. The dataset was divided into training, validation, and test sets in a **6:2:2 ratio**. Table 1 summarizes the distribution of images across the dataset, and representative samples are shown in Fig 8.

**Table 1:** Defect type composition in the PKU-Market-Phone dataset.

Defect types	Raw images	Augmentation images	Number of images
Oil	400	200	600
Scratch	400	200	600
Stain	400	200	600

### Experimental setup

To validate the effectiveness of the modules in the LNB-YOLO network, 12 groups of experiments were designed. The experimental configurations are as follows:

1. **Baseline Model:** The original YOLOv8 model was used as the baseline for the ablation study.
2. **CGRFPN Structure:** Introduced the CGRFPN structure to extract effective feature map information and enhance the detection capability for small targets.
3. **P2 Detection Layer:** Added a P2 layer as an output to further improve the detection of small targets.
4. **P6 Detection Layer:** Added a P6 layer as an output to strengthen the detection capability for large targets.
5. **LSDECD Detection Head:** Employed the LSDECD detection head to enhance the model’s ability to capture detailed features and improve large target detection accuracy.
6. **ECA Attention Mechanism:** Integrated the Efficient Channel Attention(ECA) mechanism into the network.
7. **SE Attention Mechanism:** Incorporated the Squeeze-and-Excitation(SE) attention mechanism.
8. **SimAM Attention Mechanism:** Added the Simple and Effective Attention Mechanism(SimAM).
9. **ELA Attention Mechanism:** Integrated the Efficient Local Attention(ELA) mechanism to enhance the detection performance, particularly for small defect targets.
10. **CGRFPN and ELA:** Simultaneous integration of the CGRFPN structure and the ELA attention mechanism.
11. **CGRFPN and LSDECD:** Simultaneous integration of the CGRFPN structure and the LSDECD detection head.
12. **LSDECD and ELA:** Simultaneous integration of the LSDECD detection head and the ELA attention mechanism.

The experimental results are presented in Table 2, where Group 1 represents the detection performance of the baseline YOLOv8 network model.

**Table 2:** Comparison of results of LNB-YOLO network structure ablation experiment.

Groups	CGRFPN	P2 layer	P6 layer	LSDECD	ECA	SE	SimAM	ELA	Parameter (M)	FLOPs (640@B)	mAP@0.5 (%)	mAP@.5:.95 (%)
1	-	-	-	-	-	-	-	-	3.0	8.1	91.4	59.5
2	✓	-	-	-	-	-	-	-	3.4	8.2	96.2	64.3
3	-	✓	-	-	-	-	-	-	2.9	12.4	92.5	61.3
4	-	-	✓	-	-	-	-	-	4.8	8.2	91.8	59.6
5	-	-	-	✓	-	-	-	-	2.6	5.4	95.4	62.7
6	-	-	-	-	✓	-	-	-	3.0	8.2	92.3	60.2
7	-	-	-	-	-	✓	-	-	3.0	8.2	92.5	60.8
8	-	-	-	-	-	-	✓	-	3.0	8.2	92.4	60.3
9	-	-	-	-	-	-	-	✓	3.6	8.2	96.1	63.2
10	✓	-	-	-	-	-	-	✓	4.0	8.3	96.2	66.4
11	✓	-	-	✓	-	-	-	-	2.8	6.7	96.3	65.9
12	-	-	-	✓	-	-	-	✓	3.0	6.6	95.8	65.6

**CGRFPN Module (Group 2):** In Group 2, the CGRFPN module was incorporated. Compared to the baseline, mAP@0.5 and mAP@.5:.95 improved by 4.8%, demonstrating the module’s effectiveness in detecting defect targets.

**Addition of Detection Layers (Groups 3 and 4):** Groups 3 and 4 introduced a small-target detection layer (P2) and a large-target detection layer (P6), respectively. However, despite increases in model parameters and FLOPs, no significant improvements in detection performance were observed. Consequently, the LNB-YOLO model design excludes the addition of detection layers.

**LSDECD Lightweight Detection Head (Group 5):** In Group 5, the LSDECD lightweight detection head was applied, resulting in improvements of 4.0% and 3.2% in mAP@0.5 and mAP@.5:.95, respectively. More importantly, compared to the baseline, the number of parameters and FLOPs decreased by 13.3% and 33.3%, respectively, highlighting the efficiency of the LSDECD module.

**Attention Mechanisms (Groups 6 to 9):** Groups 6 to 9 introduced various attention mechanisms into the C2FATT module within the Backbone. Among them, the ELA attention mechanism (Group 9) achieved the best detection performance, with mAP@0.5 and mAP@.5:.95 increasing by 4.7% and 3.7%, respectively.

**Final Model Design:** Based on these results, The final LNB-YOLO model was built around three core components: the CGRFPN module, the LSDECD lightweight detection head, and the ELA attention mechanism.

After determining the primary modules, we conducted Experiments 10, 11, and 12, which revealed that combining different modules significantly improved the overall detection performance of the LNB-YOLO model. This confirmed the positive synergistic effect among the modules.

After finalizing the main framework of the model, several loss functions were compared and validated, with the CIoU loss function serving as the baseline for the ablation study. As shown in Table 3, using the MDPIoU loss function improved the model’s mAP@0.5 and mAP@.5:.95 by 2.4% and 2.5%, respectively. This demonstrates that adopting the MDPIoU loss function enhances the model’s ability to detect small targets effectively.

**Table 3:** Comparison of experimental results of LNB-YOLO network with different loss functions.

Evaluation Index	CIoU Loss	DIoU Loss	EIoU Loss	GIoU Loss	WIoU Loss	SIoU Loss	MDPIoU Loss
mAP@0.5(%)	94.0	93.4	94.0	94.4	92.3	93.4	96.4
mAP@.5:.95(%)	66.7	65.8	66.1	66.5	65.1	65.8	69.2

### Comparison of experimental results of algorithm performance

To validate the performance of the LNB-YOLO network, three classic object detection algorithms—CenterNet, Faster R-CNN, SSD, and six YOLO series models (YOLOv5, YOLOv7, YOLOv8, YOLOv9, YOLOv10, and YOLOv11)—along with the latest RT-DETR model were selected for comparative evaluation. The experimental results, as shown in Table 4, indicate that the LNB-YOLO algorithm achieved the highest mAP@0.5 and mAP@.5:.95 values among all tested algorithms. This fully demonstrates the superiority of the LNB-YOLO model in detecting surface defects on mobile phones. For extremely small target detection categories, such as the Stain class, the LNB-YOLO algorithm demonstrated the best performance. These results highlight the superior small-target detection capabilities of the proposed model compared to the other ten detection algorithms.

**Table 4:** Comparison of detection results of various algorithms on the dataset.

Model Types	Pretrained weights	Parameter (M)	BFLOP/S (G)	Oil AP@0.5(%)	Scratch AP@0.5(%)	Stain AP@0.5(%)	mAP@0.5 (%)	mAP@.5:.95 (%)
YOLOv11 [43]	YOLOv11n	2.6	6.3	98.2	98.5	80.1	92.3	60.4
YOLOv10 [44]	YOLOv10n	8.1	24.8	97.4	98.3	76.5	90.7	58.6
YOLOv9 [45]	YOLOv9n	9.9	40.6	96.3	97.2	79.3	90.9	58.8
YOLOv8 [46]	YOLOv8n	3.0	8.1	97.7	99.3	77.1	91.4	59.5
YOLOv7 [47]	YOLOv7n	37.2	105.2	95.2	96.4	85.3	92.3	60.7
YOLOv5 [48]	YOLOv5n	7.3	17.0	95.4	96.8	82.6	91.6	59.2
RT-DETR [31]	rtdetr_r18vd (backbone=resnet18)	20.0	57.0	95.8	96.4	85.5	92.6	61.6
SSD [49]	SSD300	26.3	62.7	52.3	61.2	61.2	60.3	32.1
Faster R-CNN [22]	Faster-RCNN (backbone=resnet50)	137.1	370.2	45.3	57.0	57.0	54.4	30.7
CenterNet [50]	CenterNet (backbone=resnet50)	32.7	70.2	40.3	56.0	56.0	52.7	30.3
LNB-YOLO (our)	**YOLOv8n**	**3.4**	**6.7**	**99.0**	**99.5**	**90.5**	**96.4**	**69.2**

### LNB-YOLO model lightweight and knowledge distillation experiment results

Although the LNB-YOLO model demonstrated excellent detection performance on the PKU-Market-Phone dataset, its Parameters (3.4M) and FLOPs (6.7G) were 30.8% and 6.3% higher, respectively, than the latest YOLOv11 model. As a result, lightweight optimization of the LNB-YOLO model is critical to reducing inference time. To achieve this, five pruning methods were evaluated: L1 Norm Pruning (LNP), Network Slimming Pruning (NSP), Group Taylor Pruning (GTP), Layer-Adaptive Magnitude-Based Pruning(LAMP), and Dependency Graph Pruning(DGP). A fixed pruning rate of 50.0% was applied, and performance metrics were analyzed to identify the optimal method.

As shown in Table 5, the LAMP pruning method delivered the best performance, improving mAP@0.5 and mAP@.5:.95 by 0.5% and 1.5%, respectively, while reducing Parameters by 67.6% and FLOPs by 41.8%. Based on these results, LAMP was selected for pruning the LNB-YOLO model.

**Table 5:** Performance of LNB-YOLO model with different pruning methods.

Network Model	Pruning Method	Pruning Rate(%)	Knowledge Distillation	Parameter(M)	BFLOP/S(G)	mAP@0.5(%)	mAP@.5:.95(%)
LNB-YOLO	×	×	×	3.4	6.7	96.4	69.2
LNB-YOLO	LNP [51]	50%	✓	2.0	3.9	93.4	63.8
LNB-YOLO	NSP [52]	50%	✓	2.8	6.7	96.2	68.1
LNB-YOLO	GTP [53]	50%	✓	1.3	3.9	96.0	66.5
LNB-YOLO	**LAMP** [40]	50%	✓	**1.1**	**3.9**	**96.9**	**70.7**
LNB-YOLO	DGP [54]	50%	✓	2.2	3.9	96.0	67.3

After determining the pruning method, further steps were taken to optimize the pruning rate:

1. **Global Pruning:** The LAMP method was used to globally prune the LNB-YOLO model, reducing inference time.
2. **Knowledge Distillation:** The pruned LNB-YOLO model underwent knowledge distillation to preserve detection accuracy.
3. **Pruning Rate Comparison:** Post-distillation models with varying pruning rates were compared to identify the optimal rate.

The experimental results, as summarized in Table 6, show that at a pruning rate of 66.7%, the LNB-YOLO model achieved a 82.4% reduction in Parameters and a 55.2% reduction in FLOPs compared to the unpruned model. Compared to the model with a pruning rate of 50.0%, these metrics further decreased by 45.5% and 23.1%, respectively. Notably, at a pruning rate of 66.7%, mAP@0.5 improved by 1.1% compared to the unpruned model and by 0.6% compared to the 50.0% pruned model. These results indicate that the model’s complexity and inference time were significantly optimized while maintaining robust detection performance. Therefore, a pruning rate of 66.7% was selected as the final configuration for the LNB-YOLO model.

**Table 6:** Experimental results of global pruning and knowledge distillation performance of the model.

Network Model	Pruning Rate(%)	Knowledge Distillation	Parameter (M)	BFLOP/S(G)	mAP@0.5(%)	mAP@.5:.95(%)
LNB-YOLO	×	×	3.4	6.7	96.4	69.2
LNB-YOLO	50.0%	✓	1.1	3.9	96.9	70.7
LNB-YOLO	**66.7%**	✓	0.6	3.0	**97.5**	**68.8**
LNB-YOLO	75.0%	✓	0.5	2.5	92.3	61.1
LNB-YOLO	80.0%	✓	0.4	2.3	86.1	56.2

To further validate that the performance improvement of the LNB-YOLO model over the original YOLOv8 in smartphone surface defect detection is systematic rather than incidental, we employed the Wilcoxon signed-rank test for statistical analysis. Specifically, we used a test dataset containing 120 smartphone surface defect images and evaluated both models under two metrics: mAP@0.5 and mAP@0.5:.95 (%). The IoU threshold range was set between 0.5 and 0.95 with a step size of 0.05, resulting in 10 paired evaluation points for the Wilcoxon signed-rank test. Based on the paired results, we computed the difference values and corresponding p-values, as shown in Table 7. The final results demonstrated that for both mAP@0.5 and mAP@0.5:.95 (%), the p-value was 0.002. Since this value is less than 0.01, the improvements were statistically significant, thereby confirming that the performance gains of the LNB-YOLO model over the baseline YOLOv8 model are highly significant and not due to random variation.

**Table 7:** Results of the Wilcoxon signed-rank test comparing the LNB-YOLO and YOLOv8 models on the mAP@0.5 and mAP@0.5:0.95 metrics.

Network Model	mAP	IoU (0.5)	IoU (0.55)	IoU (0.6)	IoU (0.65)	IoU (0.7)	IoU (0.75)	IoU (0.8)	IoU (0.85)	IoU (0.9)	IoU (0.95)
LNB-YOLO	mAP@0.5	97.9	97.8	97.7	97.6	97.5	97.4	97.0	96.8	95.4	87.1
	mAP@.5:.95	68.2	68.6	68.9	68.8	68.8	68.7	68.5	68.4	67.4	61.2
YOLOv8	mAP@0.5	91.7	91.7	91.7	91.5	91.4	91.2	90.4	88.7	83.5	63.6
	mAP@.5:.95	59.2	59.6	59.5	59.5	59.5	59.6	59.3	58.5	55.2	41.1
P-value	mAP@0.5	P=0.002<0.01(The statistical significance threshold was set at P<0.01)									
	mAP@.5:.95										

**Fig 9:**
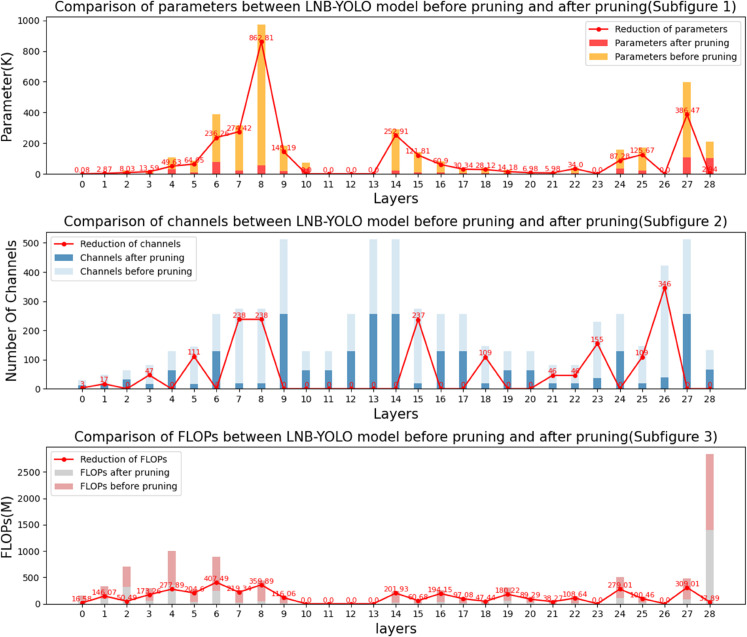
Analysis of the LAMP pruning algorithm results. The image demonstrates the LAMP pruning effect on LNB-YOLO, showing significant reductions in parameters, channels, and FLOPs across layers, which lowers computational complexity while maintaining detection performance.

Fig 9 illustrates the changes in parameter count, channel count, and FLOPs of the LNB-YOLO model after applying the LAMP pruning algorithm.

In Subfigure 1, the orange and red bar charts represent the number of parameters in each layer before and after pruning, respectively, while the red line indicates the number of pruned parameters per layer. It is evident that the parameter count in layers 6, 8, 14, and 27 decreased significantly, with an overall reduction of 82.4%.

In Subfigure 2, the light blue and dark blue bar charts depict the number of channels in each layer before and after pruning, respectively, while the red line represents the number of pruned channels per layer. The comparison reveals a substantial number of redundant channels in the original model. The LAMP algorithm effectively removes these redundant channels, as observed in layer 26, where the number of channels is reduced from 384 to 38 after pruning.

In Subfigure 3, the pink bar chart and grey bar charts represent the FLOPs in each layer before and after pruning, respectively, while the red line indicates the value of pruned FLOPs per layer. The most notable decrease occurs in layer 6, with a reduction of $407.49\times 10^{6}$ operations, while other layers also exhibit noticeable FLOPs reductions.

Overall, the LAMP pruning algorithm successfully reduces computational complexity and memory consumption by decreasing parameter count, eliminating redundant channels, and lowering FLOPs per layer, all while maintaining the model’s detection performance. This optimization provides critical support for deploying the model on cost-effective edge devices.

To evaluate the inference speed of the final LNB-YOLO model, the pre-lightweight LNB-YOLO model and the YOLOv8 model, a Frame Per Second (FPS) comparison was conducted. The calculation formula is shown in Eq (25):

$$FPS=\frac{1000}{\left(T_{pve}+T_{i}+T_{post}\right)}$$ (25)

Here, *FPS* represents frames per second (*f*/*s*), *T*_*pre*_ denotes preprocessing time, *T*_*i*_ is the inference time, and *T*_*post*_ is the postprocessing time per image.

The results, presented in Table 8, indicate that at a Batch Size of 1, the FPS ratios of the final LNB-YOLO model to the pre-lightweight LNB-YOLO model and YOLOv8 model were **1.32** and **1.02**, respectively. However, when the Batch Size increased to 64, the FPS ratios expanded to **1:1.67** and **1:1.50**, respectively. These results demonstrate that the final LNB-YOLO model achieves the fastest inference speed, especially when processing large batches of input samples.

**Table 8:** Comparison of FPS of three models.

Device	Batch Size	FPS(warmup epochs=100)		
		YOLOv8	LNB-YOLO (Original model)	LNB-YOLO (Final model)
NVIDIA GeForce RTX 3090 (24575MiB)	1	**93.6**	**72.1**	**95.2**
	2	137.7	113.4	151.5
	4	157.7	143.4	236.7
	8	171.5	163.4	302.6
	16	191.7	172.9	309.3
	32	201.2	183.2	311.3
	64	**210.5**	**189.1**	**316.5**

### Deployment and generalization

#### LNB-YOLO model edge computing deployment and test results.

The RK3568 edge computing board from Rockchip was employed for the edge deployment and application of the LNB-YOLO object detection model. The trained LNB-YOLO model weights (.pt format) were first exported as intermediate .onnx files on the host system and subsequently converted to .rknn files within the development board’s Ubuntu environment for model inference. To validate the detection performance on the edge device, the PHONE_DATASET was utilized, comprising 20 phone surface defect images containing a total of 77 targets: 16 Oils, 38 Scratches, and 23 Stains.

**Fig 10:**
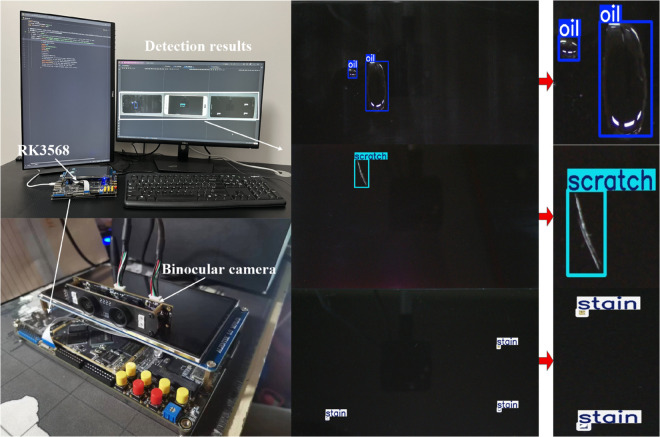
LNB-YOLO model edge computing deployment platform. The image shows the edge deployment setup and its corresponding inference results, demonstrating the practical application and real-time performance of the LNB-YOLO model in an industrial environment.

The edge deployment setup and inference results are shown in Fig 10, with the YOLOv8 model used as the benchmark for performance validation. Experimental results are summarized in Tables 9, 10, and 11.

**Table 9:** Detection results of two models in PHONE_DATASET.

Network Model	Defect Types	Type of samples			
		TP Value	FP Value	FN Value	TN Value
YOLOv8	Oil	13	3	3	52
	Scratch	38	11	0	27
	Stain	14	2	9	51
LNB-YOLO	Oil	16	1	0	58
	Scratch	38	0	0	36
	Stain	21	2	2	54

**Table 10:** Comparison of detection performance of two models for each defect category in PHONE_DATASET.

Network Model	Defect Types	Type of samples				
		PPV	FPR	FNR	TPR	F1-Score
YOLOv8	Oil	81.3%	5.5%	**18.8%**	81.3%	81.3%
	Scratch	77.6%	**28.9%**	0%	100%	87.4%
	Stain	87.5%	3.8%	**39.1%**	60.9%	71.8%
LNB-YOLO	Oil	94.1%	1.7%	**0%**	100%	97.0%
	Scratch	100%	**0%**	0%	100%	100%
	Stain	87.0%	3.6%	**8.7%**	91.3%	89.1%

**Table 11:** Comparison of overall detection performance of the two models in PHONE_DATASET.

Network Model	Method	PPV (%)	FPR (%)	FNR (%)	TPR (%)	F1-Score (%)
YOLOv8	Macro	82.1	12.7	19.3	80.7	80.2
LNB-YOLO	average	**93.7**	**1.8**	**2.9**	**97.1**	**95.4**

**Detection Accuracy (Table 9):**

1. YOLOv8 exhibited a high false-positive (FP) value for the **Scratch** defect type, indicating a high probability of misclassifying other defects as Scratches.
2. Additionally, the false-negative (FN) value for the **Stain** defect type was significantly high, revealing YOLOv8’s tendency to miss detecting Stain defects.
3. The LNB-YOLO model effectively addressed these issues, reducing both Scratch misclassification and Stain missed detections.

**Error Metrics (Table 10):**

**Fig 11:**
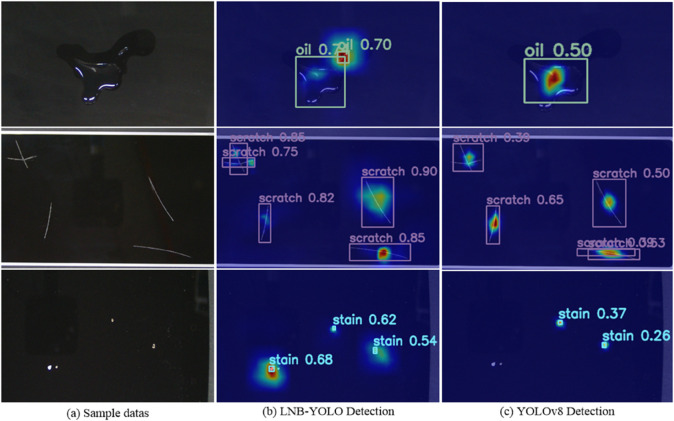
Comparison of detection effect between LNB-YOLO model and YOLOv8 model based on RK3568 board. The image shows LNB-YOLO successfully addresses missed Oil and Stain detections and reduces false positives for Scratches.

1. Compared to YOLOv8, the LNB-YOLO model achieved the following reductions:
  1). **FNR** for Oil defects: **18.8%**
  2). **FNR** for Stain defects: **30.4%**
  3). **FPR** for Scratch defects: **28.9%**
2. As shown in Fig 11**(b)** and Fig 11**(c)**, the LNB-YOLO model resolved the missed detections for Oil and Stain defects and reduced false positives for Scratch defects.

**Precision and Recall (Table 11):**

1. The LNB-YOLO model showed significant improvements over YOLOv8:
  1). **PPV**: increased by 11.6%
  2). **TPR**: increased by 16.4%
  3). **FPR**: decreased by 10.9%
  4). **FNR**: decreased by 16.4%
2. Notably, the **F1-Score** of the LNB-YOLO model reached **95.4%**, demonstrating excellent robustness and meeting industrial inspection requirements.

These results underscore the superior detection accuracy, reduced false positives and negatives, and enhanced robustness of the LNB-YOLO model, making it highly suitable for practical industrial defect detection.

### Generalization verification of LNB-YOLO model

To validate the generalization capability of the LNB-YOLO model in broader industrial scenarios, the publicly available PKU-Market-PCB dataset from Peking University’s Intelligent Robotics Open Laboratory was selected for testing. This dataset consists of 1,386 PCB images containing six defect categories: missing holes, mouse bites, open circuits, short circuits, spurious copper, and spurs. The dataset was divided into training, validation, and testing sets in a 6:2:2 ratio.

**Fig 12:**
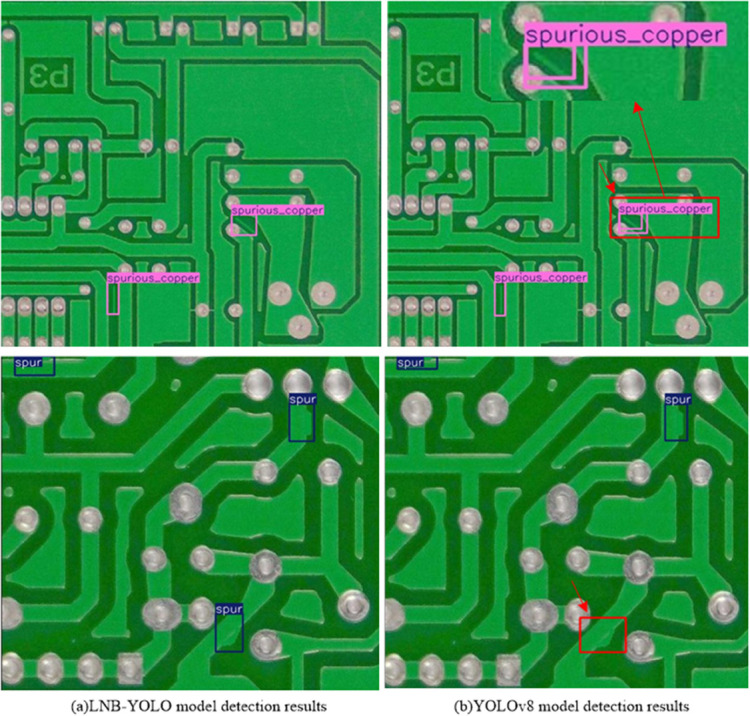
Comparison of defect identification performance results of LNB-YOLO and YOLOv8 models in PKU-Market-PCB. The image shows LNB-YOLO outperforms YOLOv8, increasing mAP scores and eliminating overlapping and missed detections for spurious copper and spur defects.

The detection results are illustrated in Fig 12 and summarized in Table 12. Compared to YOLOv8, the LNB-YOLO model achieved improvements of 3.0% and 11.8% in mAP@0.5 and mAP@.5:.95, respectively, while maintaining robust floating-point performance. Notably, the LNB-YOLO model avoided overlapping detection boxes and missed detections for the spurious copper and spur categories, as shown in Fig 12(a). In contrast, YOLOv8 exhibited overlapping detection boxes and missed detections for these targets, as illustrated in Fig 12(b). These results demonstrate the excellent generalization capability of the proposed LNB-YOLO model.

**Table 12:** The overall detection performance of two models in PKU-Market-PCB.

Network Models	Parameter (M)	BFLOP/S (G)	mAP@0.5 (%)	mAP@.5:.95 (%)
YOLOv8	3.0	8.1	79.7	40.8
LNB-YOLO	3.4	6.7	82.7	52.6

## Conclusion and future work

This paper addresses the challenges of detecting small defects on mobile phone screens by proposing a lightweight detection network, LNB-YOLO, based on improvements to key components of YOLOv8. First, the CGRFPN structure is introduced in the Head section to enhance the model’s ability to perceive features at different levels and detect targets in complex backgrounds. Second, the ELA attention module is integrated into the C2F module of the Backbone to comprehensively improve the feature localization capability. The MPDIoU loss function replaces the original CIoU loss function to prevent gradient explosion issues. Additionally, the LSDECD lightweight detection head is designed to further enhance the model’s ability to capture small targets. Model pruning and knowledge distillation are employed to reduce the complexity and computational cost of the LNB-YOLO model. Finally, the RK3568 AI development board is used to deploy the LNB-YOLO model on edge devices, to evaluate its performance in edge applications.

Experimental results demonstrate that the proposed algorithm achieves mAP@0.5 and mAP@.5:.95 of 97.5% and 68.8%, respectively, on the PKU-Market-Phone dataset, representing improvements of 6.1% and 9.3% over the original YOLOv8. Meanwhile, the parameter count decreases by 80.0%, and computational cost decreases by 63.0%.

To validate the generalization capability of the LNB-YOLO model, it was tested on datasets from other scenarios. The results show that the LNB-YOLO model achieved mAP@0.5(%) and mAP@.5:.95(%) of 82.7% and 52.6%, which are 3.1% and 4.8% higher than those of the YOLOv8 model. This demonstrates the excellent generalization capability of the LNB-YOLO model, particularly its precision and efficiency in small target detection.

This algorithm provides an accurate, efficient, and resource-optimized solution for industrial applications, making it suitable for deployment on edge terminal devices and advancing defect detection technologies. Future research could expand to cover the back and other external parts of mobile phones to enable comprehensive and precise evaluation of their appearance.
